# A Novel Intraoral Technique for Botulinum Toxin Type A Application in the Mentalis Muscle

**DOI:** 10.7759/cureus.105783

**Published:** 2026-03-24

**Authors:** Saverio Lembo, Paola Macias-Arias, Maria Paulina Uribe Posada, Raul Banegas, Jaime Rengifo

**Affiliations:** 1 Anatomy and Head and Neck Surgery, Hospital of San Miguel, Buenos Aires, ARG; 2 Dermatology, Federico Lleras Acosta ESE University Hospital Dermatological Center, Bogota, COL; 3 Dermatology, Private Practice, Medellin, COL; 4 Plastic Surgery, Private Practice, Buenos Aires, ARG; 5 Dermatology, Pontifical Bolivarian University, Medellin, COL

**Keywords:** anatomical approach, botulinum toxin type a, chin contouring, intraoral injection technique, mentalis muscle

## Abstract

The mentalis muscle plays a critical role in lower facial balance by stabilizing the lower lip and shaping the chin contour. Botulinum toxin type A (BoNT-A) is commonly used to manage excessive mentalis activity; however, inaccurate injection techniques may lead to functional impairment and undesirable aesthetic outcomes.

This technical report describes a novel and innovative intraoral botulinum toxin injection technique targeting the mentalis muscle based on precise anatomical localization of its origin. The proposed method introduces a new intraoral approach designed to improve injection accuracy while minimizing toxin diffusion and preserving lower lip function. A detailed anatomical assessment was performed to define the muscle’s origin, fiber orientation, and relevant surface landmarks, which informed the development of a controlled injection strategy emphasizing precise depth and placement.

In clinical application, the technique was performed in three patients, demonstrating consistent modulation of mentalis contraction, improvement in chin surface appearance, and preservation of lower lip function without functional compromise. This new and reproducible technique highlights the importance of anatomical precision in BoNT-A injection planning and provides a practical framework to enhance safety and aesthetic outcomes in treatments of the lower third of the face.

## Introduction

The mentalis muscle plays a significant role in facial harmonization, despite not being directly involved in oral function. It contributes to the expression of emotions such as grimacing or pouting. This muscle provides stability to the lower lip by contributing to its elevation and is responsible for the formation of chin wrinkles [1].

Botulinum toxin is commonly used to control the hyperactivity of the mentalis muscle. However, effective and safe treatment requires a precise understanding of its anatomy to avoid adverse effects such as compromised oral closure or smile asymmetry.

The aim of this article is to propose a novel intraoral neuromuscular blockade injection technique targeting the origin of the mentalis muscle. This approach seeks to enhance safety and efficacy by improving injection accuracy and minimizing associated risks. To our knowledge, this is the first report describing a targeted intraoral botulinum toxin injection technique directed at the anatomical origin of the mentalis muscle.

## Technical report

The mentalis muscle is a key structure in facial harmonization procedures. It is located in the central region of the mandible, originating from the anterior portion of the mandible and inserting into the skin of the chin. Its primary function is to elevate the soft tissue of the chin and support the position and movement of the lower lip, contributing to lower facial balance and the formation of chin wrinkles [1].

During the aging process, several anatomical and structural changes occur in this region. These include the appearance of the mental fold, which corresponds to a deep groove between the lower lip and the prominence of the chin caused by repeated contraction of the mentalis muscle; the development of a “peau d’orange” appearance, associated with the loss of subcutaneous fat and dermal collagen in the chin region; and alterations in lower facial contour when the activity of the mentalis muscle becomes unbalanced relative to surrounding tissues. Due to these factors, the mentalis muscle has gained increasing relevance in aesthetic dermatology [1,2].

Botulinum toxin is commonly used for both aesthetic and therapeutic purposes in the facial region, including the lower third of the face. However, effective treatment requires a precise understanding of the anatomy of the mentalis muscle, its variations, and its functional relationships with surrounding musculature. Our primary focus is to present a novel intraoral injection technique targeting the anatomical origin of the mentalis muscle in order to improve injection accuracy and reduce the risk of toxin diffusion to adjacent muscles.

Anatomically, the area occupied by the mentalis muscle spans approximately 5-10 mm in the sagittal midline and 20-30 mm in the horizontal dimension, with a depth ranging from 6.7 to 10.7 mm. The muscle lies at an average distance of 16.7 ± 3.8 mm from the mental foramen. The depth from the skin surface is approximately 6.7 ± 1.4 mm, with a muscle thickness of 4.0 ± 1.4 mm, and a distance of approximately 1.1 mm from the bone corresponding to the submentalis fat layer. No significant anatomical differences have been reported between genders [3,4].

**Figure 1:**
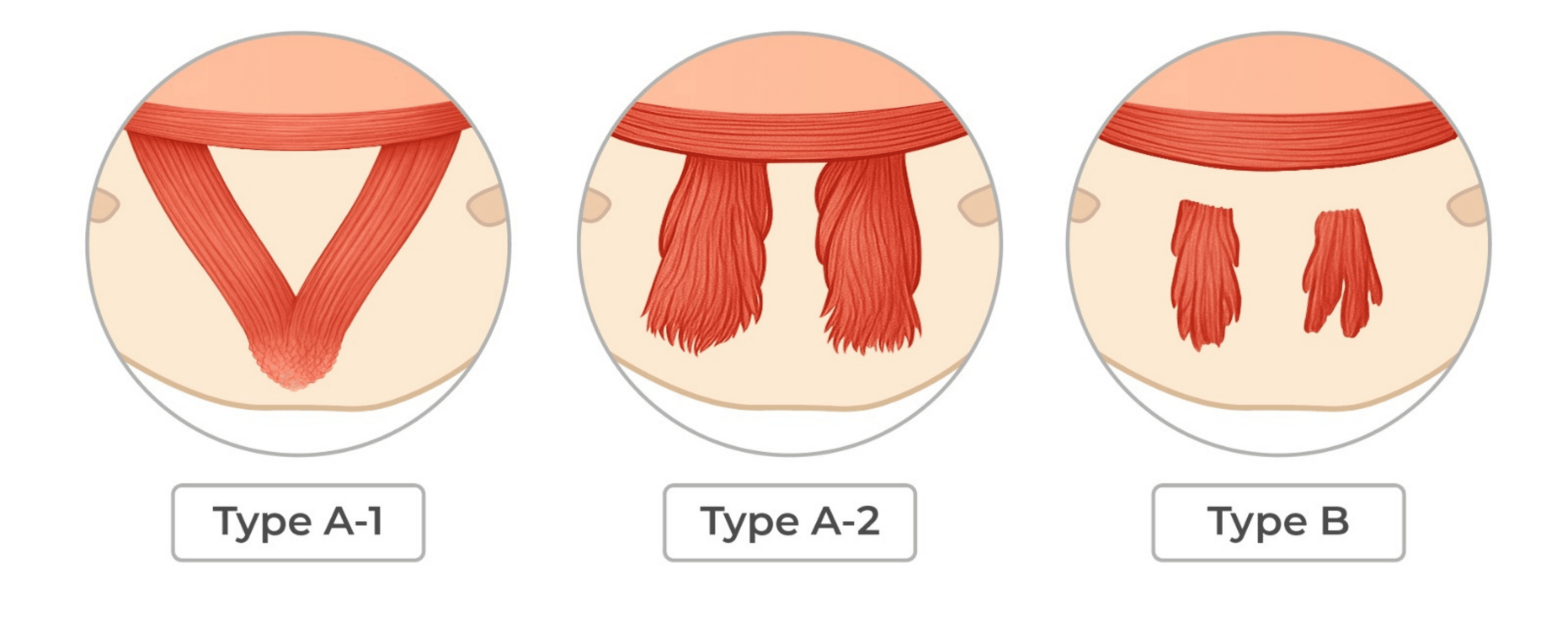
Anatomical variants illustration The figure was designed using Adobe (San Jose, CA, USA) by digital designer Kelly Medina, to whom the authors extend their special thanks, under the coordination of epidemiologist María Alejandra Palacios

Two principal anatomical variants of the mentalis muscle have been described. Type A, the most common configuration, occurs in up to 86.4% of cases and presents a three-dimensional dome-shaped structure. This type can be subdivided into type A1 (fused type), in which the muscle bellies are fused and observed in approximately 47.7% of cases, and type A2 (separated type), in which the muscle bellies remain separated, occurring in about 38.6% of cases. Type B represents a thinner, flatter configuration with separated muscle bellies and a trapezoidal shape, accounting for approximately 13.6% of cases. A summary of these variants is presented in Table 1 (see also Figure 1) [3-6].

**Table 1:** Anatomical variants of the mentalis muscle

Variant	Description	Prevalence
Type A1 (fused)	Dome-shaped muscle with fused bellies	47.7%
Type A2 (separated)	Dome-shaped muscle with separated bellies	38.6%
Type B	Flat, thinner trapezoidal muscle with separated bellies	13.6%

From an aesthetic perspective, it is essential to understand that multiple muscles may share similar cutaneous insertion points while exerting different directional vectors. These vectors maintain a dynamic equilibrium that contributes to facial expression and functional harmony. The administration of botulinum toxin can disrupt this balance if diffusion affects adjacent muscles, potentially altering the activity and positional relationships of structures in the lower third of the face [2,4-6].

**Figure 2:**
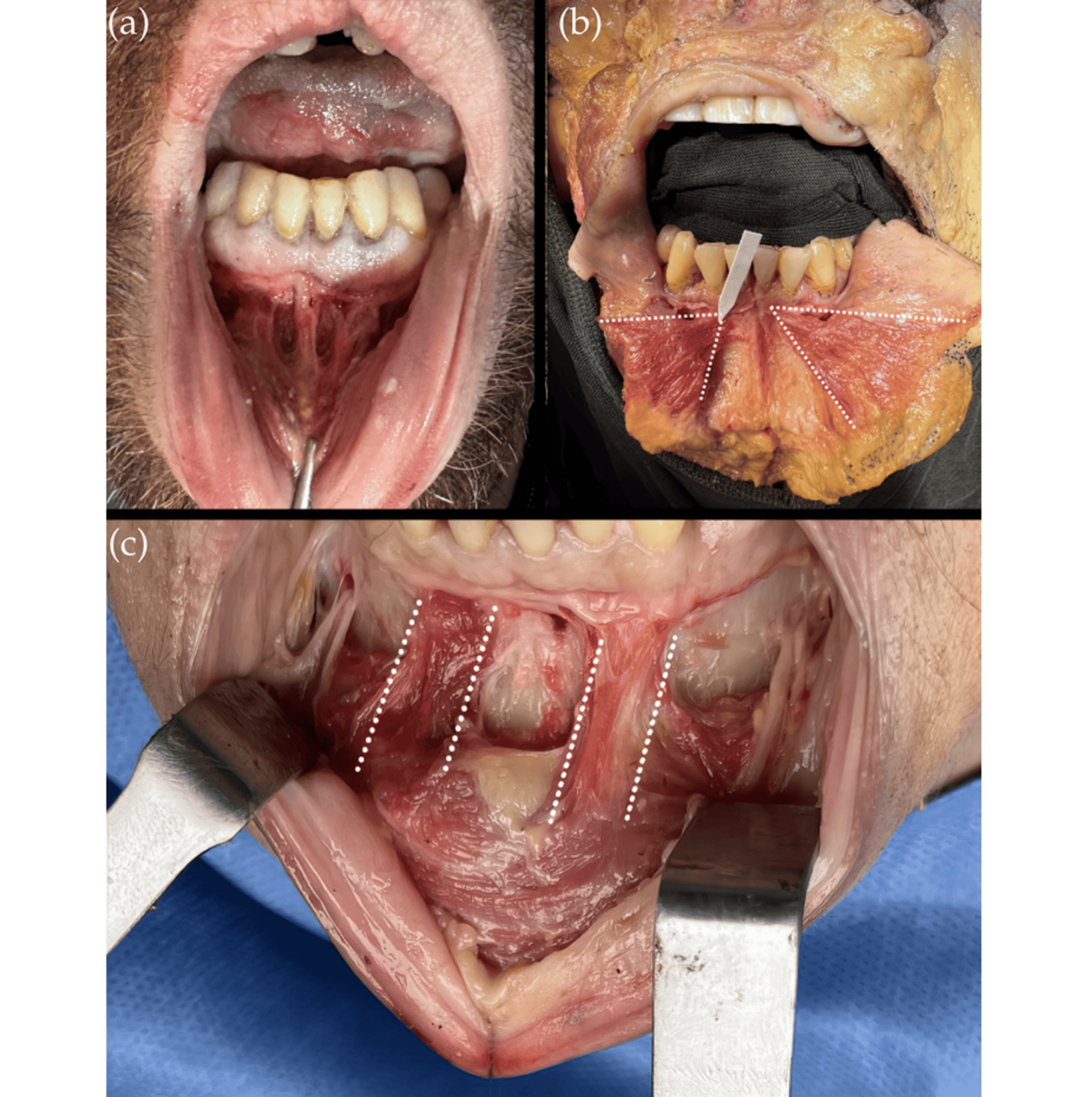
Anatomical structure and fiber orientation of the mentalis muscle (a) Intraoral view of the mentalis muscle in a dissection specimen. (b) Dissection specimen showing the mentalis muscle and its midline fibers. (c) Deep intraoral dissection highlighting the vertical fiber compartments of the mentalis muscle

The muscle fibers of the mentalis originate from the alveolar bone of the anterior mandible and insert into the dermis of the chin, typically closer to the midline than to their point of origin. As these fibers insert into the skin, contraction of the muscle produces the characteristic mental fold. The mentalis muscle is composed of medial, lateral, superior, and inferior fibers. The medial fibers, located on both sides of the midline, descend in an anteromedial dome-shaped pattern. The lateral fibers descend obliquely and insert into the ipsilateral skin, partially intermingling with the depressor labii inferioris muscle fibers. The superior fibers run horizontally and interlace with the inferior margin of the orbicularis oris muscle, contributing to lower lip elevation [4-7]. These anatomical relationships are illustrated in Figure 2.

Based on this anatomical understanding, we propose a novel intraoral injection approach directed at the anatomical origin of the mentalis muscle. This strategy aims to improve precision in botulinum toxin delivery, minimize toxin diffusion, and preserve the functional balance of the lower lip musculature.

**Figure 3:**
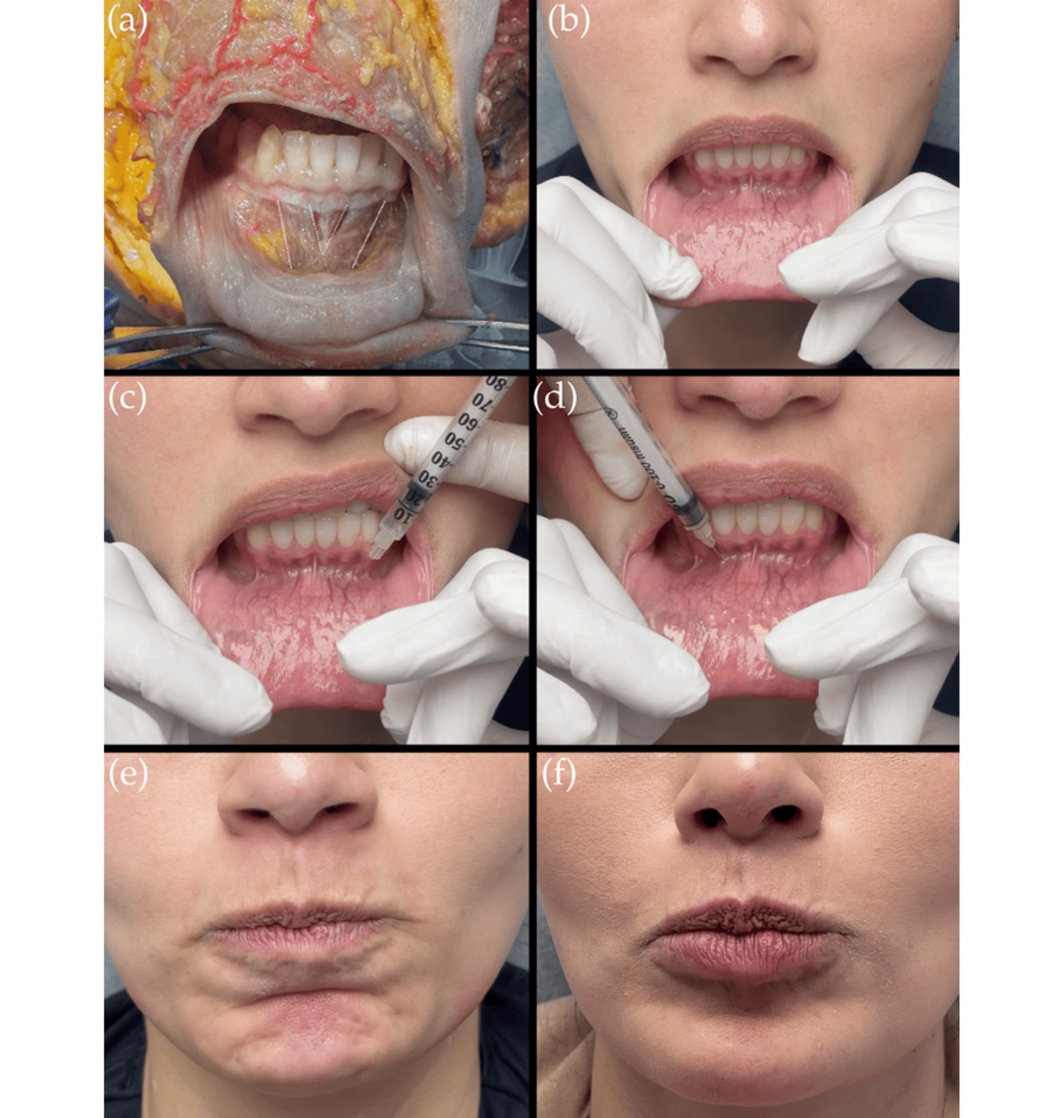
Step-by-step sequence for the intraoral administration of botulinum toxin in the mentalis muscle (a) Cadaveric dissection illustrating the intramuscular fiber orientation of the mentalis. (b) Clinical identification of the intraoral access point. (c, d) Intraoral needle insertion into the mentalis muscle. (e) Pre-treatment activation of the mentalis muscle (“pouting movement”). (f) Post-treatment relaxation of the mentalis muscle

This article proposes a novel intraoral technique for botulinum toxin type A (BoNT-A) injection targeting the origin of the mentalis muscle (Figure 3). This method allows direct application at the muscle origin, thereby reducing the risk of involvement of adjacent muscle groups.

The procedure consists of the following steps: (1) Explain the procedure to the patient and obtain signed informed consent. (2) Take pre-procedure clinical photographs. (3) Evaluate the dynamics of the muscular groups in the chin region and identify the specific contraction pattern of the mentalis muscle. (4) Prepare the necessary materials for injection and determine the appropriate BoNT-A dose. (5) Perform aseptic and antiseptic preparation of the intraoral site. (6) Evert the patient’s lower lip to expose the origin of the mentalis muscle, located at the midline just inferior to the central incisors. Inject directly at the muscle origin, between the central and lateral incisors on both sides of the lower dental arch. In dental universal nomenclature, this corresponds to the region between teeth 3.1 and 3.2, and 4.1 and 4.2, located immediately below the inferior gingivobuccal sulcus. (7) The 100 U vial was diluted in 2 mL of saline, and 2-4 U were injected per point. According to the 2022 Italian consensus by Signorini et al., recommended doses range between 4 and 5 U per injection point [8]. However, in the authors’ experience, adequate muscle blockade was achieved with the lower doses used in this technique, likely because the injection directly targets the origin of the muscle. (8) Use a cotton roll or sterile gauze to gently clean the injection area. (9) Schedule a follow-up appointment approximately two weeks after the procedure to evaluate clinical outcomes and monitor for potential complications.

To illustrate the technical application of this approach, three clinical cases are presented in which botulinum toxin was administered using the intraoral technique targeting the mentalis muscle. Pre- and post-treatment images demonstrate improved control of the muscle’s dynamic activity without documented complications or asymmetries (Figure 3). These cases are included solely to illustrate the technical application of the method and are not intended as a formal case series or outcome analysis.

**Figure 4:**
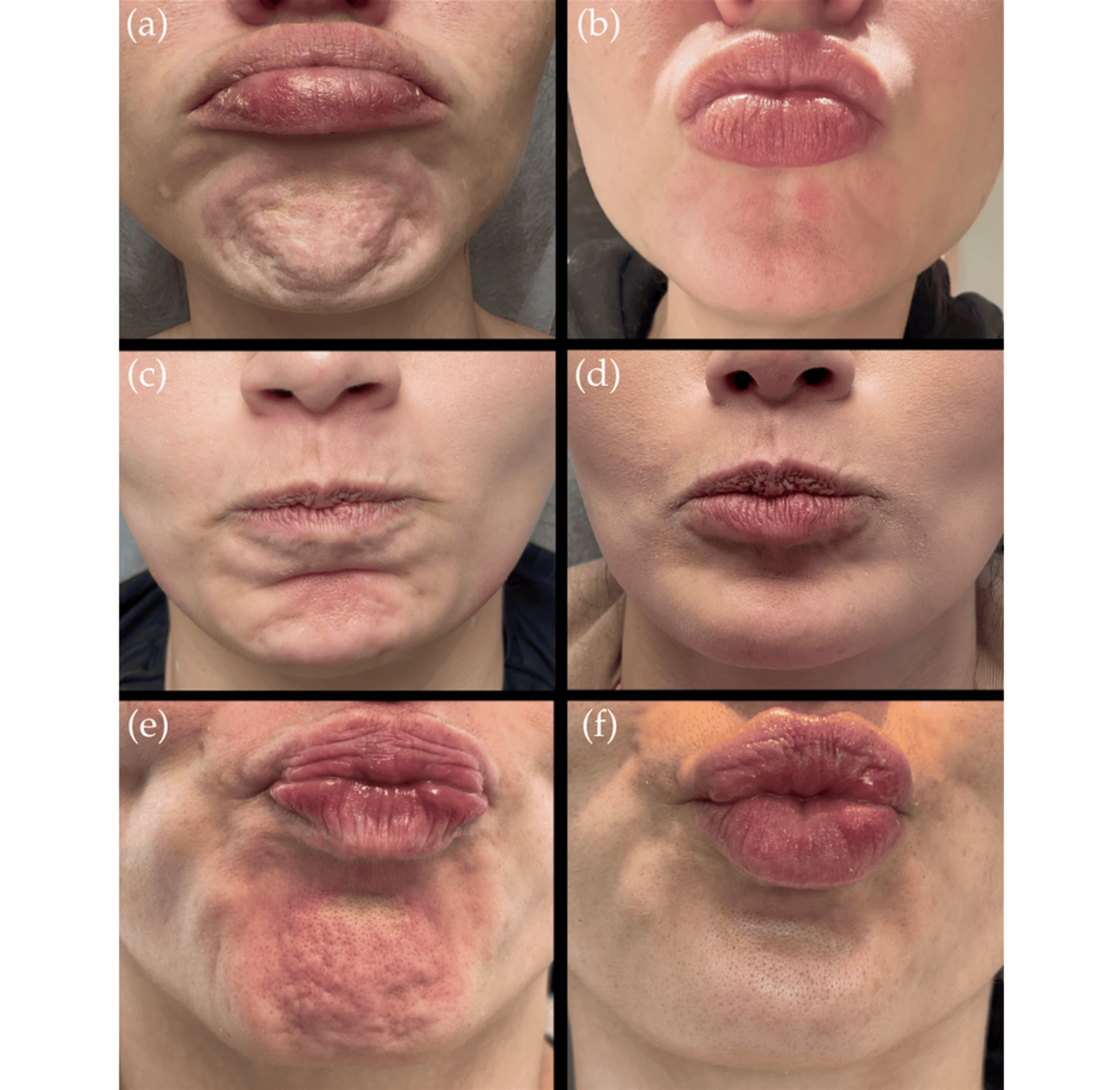
(a, c, e) Contraction of the mentalis muscle. (b, d, f) After application of botulinum toxin type A to the mentalis muscle

Below, we present three cases in which botulinum toxin was applied using the intraoral approach to the mentalis muscle. Pre- and post-treatment outcomes are shown, demonstrating optimal control of the muscle’s dynamic activity without documented complications or asymmetries (Figure 4). These cases are presented solely to illustrate the technical application and are not intended as a case series or outcome analysis.

## Discussion

The mentalis muscle plays a critical role in the dynamic positioning of the chin and lower lip, as well as in maintaining the aesthetic balance of the lower third of the face. Zide emphasized its importance as a key determinant of chin projection and lower lip posture, highlighting the potential for both functional and aesthetic disturbances when this muscle is improperly treated [1]. Consequently, BoNT-A injection into the mentalis muscle requires a precise understanding of its anatomy, depth, and relationship with adjacent musculature.

Anatomical studies have demonstrated significant variability in the morphology, fiber orientation, and insertion patterns of the mentalis muscle. Hur et al. described its close anatomical relationship with the orbicularis oris and incisivus labii inferioris muscles, highlighting the risk of toxin diffusion to adjacent structures when injections are placed too superficially or laterally [4]. These anatomical considerations are consistent with clinical references emphasizing that even minor deviations in injection depth or location may result in suboptimal outcomes or complications [2].

Several injection techniques have been proposed to address these challenges. In 2001, Le Louarn suggested medial and superficial injections in the upper region of the muscle [7]. Later, Carruthers and Carruthers proposed a safer approach involving injection sites positioned more distal to the orbicularis oris muscle [9]. Klein further emphasized the importance of precise BoNT-A administration, noting that improper technique may lead to unintended paralysis of adjacent muscles such as the depressor labii inferioris or the orbicularis oris, potentially resulting in speech difficulties, problems with eating or drinking, or facial asymmetry [10].

Recent cadaveric and ultrasonographic studies have contributed to a more refined understanding of optimal injection locations within the mentalis muscle. Choi et al. identified effective injection points corresponding to the central muscle belly, suggesting that targeting this region may improve efficacy while minimizing toxin spread [3]. Similarly, Yi et al. proposed imaging-based anatomical guidelines that emphasize individualized injection strategies rather than reliance solely on fixed surface landmarks [5].

With regard to the injection plane, anatomical studies suggest considering the distance between the mentalis muscle and the underlying mandibular bone. The optimal muscular plane has been estimated at approximately 9 mm in depth, which may improve injection accuracy. Additionally, the direction of the muscle fibers should be considered: in the superior portion, fibers run horizontally toward the anterior cutaneous surface, whereas in the inferior portion, they follow an inferomedial or vertical orientation [11]. Current recommendations for injection placement often include both deep and superficial points located approximately 0.5 cm lateral to the pogonion [12,13].

The technique presented in this report proposes a targeted intraoral approach directed at the anatomical origin of the mentalis muscle, allowing for more precise neuromuscular blockade while potentially reducing unintended toxin diffusion to adjacent muscles. By improving injection accuracy, this approach may help preserve lower lip function while enhancing aesthetic outcomes, including reduction of the characteristic “peau d’orange” appearance and softening of the mental crease. These effects highlight the importance of anatomical precision in BoNT-A administration and its potential impact on both functional preservation and aesthetic improvement in treatments of the lower third of the face.

Another clinically relevant consideration is paradoxical bulging of the mentalis muscle, a phenomenon that may occur when BoNT-A is injected in a manner that fully penetrates the muscle thickness or when toxin distribution is uneven. Ultrasonographic observations suggest that this effect results from selective weakening of superficial fibers with preservation of deeper muscle activity, leading to compensatory contraction [6]. This complication has also been described in clinical series as an underrecognized cause of suboptimal aesthetic outcomes. For this reason, in 2020, Yu et al. proposed injecting BoNT-A at multiple muscle depths to prevent paradoxical chin bulging following treatment of the mentalis muscle [12]. In patients with pre-existing hyaluronic acid filler in the chin region, a deeper and more targeted application has been suggested to reduce this risk by ensuring more homogeneous neuromuscular blockade [6,12]. While this strategy seeks to minimize aesthetic and functional complications, such as chin ptosis or lip incompetence, it also highlights the limitations of depth-based techniques. In contrast, the technique presented in this report targets the precise anatomical origin of the mentalis muscle, allowing for more accurate toxin delivery with fewer injection points. This anatomical-based approach enhances precision and control, thereby reducing the risk of adverse outcomes.

Diffusion and spread of BoNT-A remain additional concerns in the lower face, where functional impairment can significantly affect speech, mastication, and emotional expression. Prior studies have demonstrated that toxin migration is influenced by dose, concentration, injection volume, and tissue planes [7,13,14]. Carruthers and Carruthers emphasized that conservative dosing and anatomically guided placement are particularly critical in the mid and lower face to prevent adverse events such as lower lip incompetence or dysphagia [8]. The anatomically guided technique presented in this report aligns with these principles and may contribute to improved safety.

Beyond aesthetic considerations, excessive mentalis muscle activity has been associated with increased mechanical stress on the mandibular dental arch. Finite element analyses suggest that chronic mentalis overactivity may influence mandibular biomechanics, supporting the potential functional relevance of precise neuromodulation of this muscle [15].

Other important considerations include the diffusion halo of BoNT-A, which can extend from 15 to 45 mm from the injection site, depending on the type of neurotoxin, dose, and volume used. As such, a single injection point, placed at the correct location and depth, may be sufficient to neuromodulate the mentalis muscle, whose average length ranges from 18.0 ± 3.9 mm, and width from 10.9 ± 3.0 mm [13,14]. Nevertheless, clinicians may adjust the dose when necessary by evaluating muscle thickness through ultrasonography. However, it is important to note that not all clinical settings offering botulinum toxin treatment are equipped with ultrasound technology.

For the procedure, BoNT-A is typically diluted in 2.5 mL of normal saline, reconstituted to a concentration of 4 U/0.1 mL. Higher dilution volumes are discouraged, as they are associated with increased diffusion into surrounding tissues. Deep injections should be administered at 3 U, with the technique involving contacting the mandibular bone, followed by slight needle retraction before injection. Superficial injections involve 1 U administered into the subdermal plane [14,15].

This technical report has limitations. As a descriptive technique-based study, it lacks quantitative outcome measures and long-term follow-up data. Additionally, interindividual anatomical variability may limit the universal applicability of a single injection approach. Future studies incorporating ultrasonographic guidance, standardized outcome assessments, and larger patient cohorts are needed to further validate the reproducibility and clinical benefits of this technique.

In conclusion, advanced knowledge of mentalis anatomy and refined BoNT-A application techniques enable more precise, lower-risk interventions in the aesthetic management of the lower face. By targeting the muscle at its anatomical origin and accounting for known mechanisms of diffusion and paradoxical bulging, the technique described in this report offers a rational, anatomy-based strategy to improve both safety and aesthetic outcomes.

## Conclusions

A precise understanding of the anatomy, function, and anatomical variability of the mentalis muscle is essential to achieve safe and predictable outcomes in aesthetic procedures involving the lower third of the face. Recent anatomical and imaging studies have emphasized that accurate localization of the mentalis muscle and appropriate injection depth are key factors in minimizing complications and improving treatment precision. The novel intraoral injection technique proposed in this report aims to improve accuracy by targeting the anatomical origin of the mentalis muscle, potentially reducing unintended toxin diffusion and preserving lower lip function. By focusing on anatomically guided placement, this approach may enhance both safety and treatment efficacy in aesthetic dermatology.

Beyond aesthetic improvement, precise neuromodulation of the mentalis muscle may also contribute to functional balance in the lower face, including improved control of excessive muscle contraction associated with the mental crease and “peau d’orange” appearance of the chin. Although this report primarily describes a technical approach, the method may represent a promising alternative to traditional injection techniques. Further clinical studies involving larger patient cohorts and standardized outcome measures are needed to confirm the reproducibility, safety, and long-term efficacy of this intraoral technique.
